# IRF4 in Skeletal Muscle Regulates Exercise Capacity via PTG/Glycogen Pathway

**DOI:** 10.1002/advs.202001502

**Published:** 2020-08-01

**Authors:** Xiaopeng Zhu, Ting Yao, Ru Wang, Shanshan Guo, Xin Wang, Zhenqi Zhou, Yan Zhang, Xiaozhen Zhuo, Ruitao Wang, John Zhong Li, Tiemin Liu, Xingxing Kong

**Affiliations:** ^1^ Division of Pediatric Endocrinology Department of Pediatrics UCLA Children's Discovery and Innovation Institute David Geffen School of Medicine at UCLA Los Angeles CA 90095 USA; ^2^ Department of Endocrinology and Metabolism Zhongshan Hospital Fudan University Shanghai 200032 P. R. China; ^3^ Fudan Institute for Metabolic Disease Fudan University Shanghai 200032 P. R. China; ^4^ School of Kinesiology Key Laboratory of Exercise and Health Sciences of Ministry of Education Shanghai University of Sport Shanghai 200438 P. R. China; ^5^ Department of Internal Medicine Harbin Medical University Cancer Hospital Harbin Medical University No. 150 Haping ST, Nangang District Harbin Heilongjiang 150081 P. R. China; ^6^ Department of Medicine Division of Endocrinology, Diabetes and Hypertension David Geffen School of Medicine University of California Los Angeles CA 90095 USA; ^7^ School of Life Sciences Fudan University Shanghai 200032 P. R. China; ^8^ Department of Cardiology The First Affiliated Hospital Xi'an Jiaotong University Xi'an Shanxi 710061 P. R. China; ^9^ The Key Laboratory of Rare Metabolic Disease Department of Biochemistry and Molecular Biology The Key Laboratory of Human Functional Genomics of Jiangsu Province Nanjing Medical University Nanjing Jiangsu 211166 P. R. China; ^10^ State Key Laboratory of Genetic Engineering Department of Endocrinology and Metabolism and School of Life Sciences Zhongshan Hospital Fudan University Shanghai 200032 P. R. China; ^11^ Institute of Metabolism and Integrative Biology and Collaborative Innovation Center for Genetics and Development Fudan University Shanghai 200032 P. R. China; ^12^ Human Phenome Institute Fudan University Shanghai 200032 P. R. China

**Keywords:** exercise capacity, glycogen, IRF4, PTG, skeletal muscle

## Abstract

Exercise‐induced fatigue and exhaustion are interesting areas for many researchers. Muscle glycogen is critical for physical performance. However, how glycogen metabolism is manipulated during exercise is not very clear. The aim here is to assess the impact of interferon regulatory factor 4 (IRF4) on skeletal muscle glycogen and subsequent regulation of exercise capacity. Skeletal muscle‐specific IRF4 knockout mice show normal body weight and insulin sensitivity, but better exercise capacity and increased glycogen content with unaltered triglyceride levels compared to control mice on chow diet. In contrast, mice overexpression of IRF4 displays decreased exercise capacity and lower glycogen content. Mechanistically, IRF4 regulates glycogen‐associated regulatory subunit protein targeting to glycogen (PTG) to manipulate glucose metabolism in skeletal muscle. Knockdown of PTG can reverse the effects imposed by the absence of IRF4 in vivo. These studies reveal a regulatory pathway including IRF4/PTG/glycogen synthesis on controlling exercise capacity.

## Graphical abstract

Muscle glycogen is critical for exercise‐induced fatigue. However, how glycogen is manipulated during exercise is not well‐known. Interferon regulatory factor 4 (IRF4) was induced by exercise. Mice without IRF4 specific in muscle show increased glycogen content pre‐exercise and subsequently affects exercise capacity. These studies reveal a feedback signaling pathway including IRF4/protein targeting to glycogen (PTG)/glycogen synthesis that controlling exercise capacity.

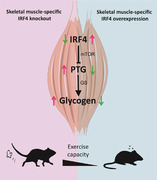

## 1 Introduction

Muscle glycogen synthesis comprises a principal pathway of glucose storage. Impairment of muscle glucose uptake and glycogen synthesis are major contributors to insulin resistance and type 2 diabetes mellitus (T2DM).^[^ ^1^ ^]^ Results also suggest that muscle glycogen availability can affect performance during both short‐term and more prolonged high‐intensity intermittent exercise.^[^ ^2^ ^]^ One of the first studies using the needle biopsy technique to obtain samples of human skeletal muscle showed a marked muscle glycogen depletion after prolonged (≈2 h) cycling exercise, and the time until exhaustion was correlated to the pre‐exercise glycogen concentration.^[^ ^3^ ^]^ Since then, many studies have confirmed that exercise capacity is severely compromised when muscle glycogen content is depleted to very low levels.^[^ ^4^, ^5^ ^]^ However, the exact mechanism(s) by which causes glycogen depletion remains uncertain.

Glycogen synthesis depends on the activity of glycogen synthase (GS) and protein phosphatase 1 (PP1). For glycogen synthesis to occur, GS and PP1 must localize to glycogen particles, which occurs through glycogen‐associated regulatory subunits (PP1 G‐subunits). PP1 G‐subunits constitute a family of proteins including PPP1R3A, PPP1R3B, PPP1R3C, PPP1R3D, and PPP1R3E. G‐subunits display tissue‐ and species‐specific expression. In rodent skeletal muscle, several members of the G‐subunits gene family are expressed; the muscle‐specific PPP1R3A and PPP1R3C (encoding protein named protein targeting to glycogen (PTG)), which are expressed mostly in muscle and liver; and the relatively wide spread isoform PPP1R3D. PPP1R3B^[^ ^6^ ^]^ and PPP1R3E^[^ ^7^ ^]^ genes are not significantly expressed in rodent. Among them, PTG plays a crucial role in controlling glycogen synthesis,^[^ ^8^, ^9^, ^10^ ^]^ and its overexpression dramatically increases glycogen content because of a redistribution of PP1 and GS to glycogen particles.^[^ ^8^, ^9^, ^11^, ^12^ ^]^ However, the upstream of PTG is rarely investigated.

Rosen and his co‐workers identified IRF4 as an antiadipogenic transcription factor following a screen based on epigenomic changes during differentiation.^[^ ^13^ ^]^ Our previous work suggested that IRF4 serves as a key molecular node in virtually all of the metabolic actions of adipose tissue. Specifically, we have addressed that IRF4 regulates lipolysis and lipogenesis in white and brown adipocytes, thermogenesis in brown adipocytes, and M2 polarization in adipose‐resident macrophages.^[^ ^14^, ^15^ ^]^ IRF4 is regulated transcriptionally by nutritional state and cold exposure, and directly interacts with co‐factors such as PGC‐1*α* in brown fat. PGC‐1*α* can regulate metabolic genes in skeletal muscle and contributes to the response of muscle to exercise. Recently, there are some studies showing a link between exercise and thermogenesis. In terms of this, we propose to delve more deeply into the mechanisms by which IRF4 exerts its metabolic actions in skeletal muscle.

To investigate the role of IRF4 in skeletal muscle tissue, we generated mouse lines in which loss‐ or gain‐of‐function of IRF4 was induced in a skeletal muscle‐specific manner. Here, we described that muscle‐specific IRF4 deletion (muscle‐specific IRF4 knockout (MI4KO)) increased glycogen content in skeletal muscle prior to exercise when compared to wild‐type (WT) mice on chow diet. Conversely, mice that overexpressed IRF4 transgenically in skeletal muscle displayed decreased exercise capacity. Most importantly, within the skeletal muscle, loss of IRF4 caused upregulation of PTG expression and, subsequently, increased GS protein level. Finally, knockdown of PTG can block the effects imposed by the absence of IRF4, further showing the role of IRF4 in regulating PTG expression. Collectively, these studies indicate that IRF4 is a coordinator of glycogen synthesis via regulation of PTG.

## 2 Results

### 2.1 IRF4 Expression Is Regulated by Exercise in Skeletal Muscle

**Figure 1:**
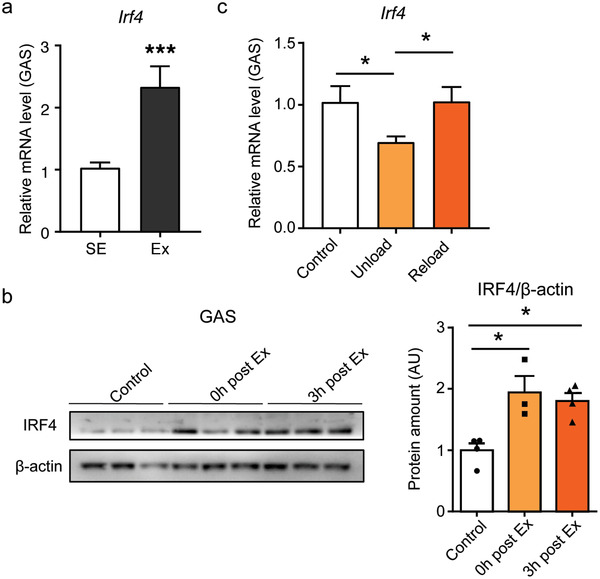
The association of IRF4 in skeletal muscle with exercise. a) qPCR analysis of Irf4 expression in skeletal muscle of mice in sedentary and exercise groups (*n* = 7, ***p < 0.001). Male C57BL/6J wid‐type (WT) mice were individually housed in cages with access to a running wheel (free wheel‐running) for 30 days and sacrificed after 1 h treadmill exercise. b) Western blot analysis of the expression of IRF4 in skeletal muscle of WT mice in sedentary, 0 h after exercise, and 3 h after exercise group. Protein amount was quantified using ImageJ (*n* = 3–4, *p < 0.05). c) qPCR analysis of Irf4 expression in skeletal muscle of WT mice in control (nonsuspension), unload (suspension for 7 days), and reload (7 days reload after 7 days suspension) (*n* = 4, *p < 0.05). All results are expressed as means ± SEM.

We first addressed whether skeletal muscle IRF4 expression was regulated by exercise. Indeed, Irf4 expression was upregulated after exercise in skeletal muscle of trained mice (**Figure** **1**a). IRF4 expression was also measured in skeletal muscle from mice with acute exercise compared to sedentary mice. Its expression was increased even after one bout exercise (Figure 1b). It is interesting that the expression of IRF4 was also increased in adipose tissue and heart after exercise (Figure S1a,b, Supporting Information), indicating that the increased expression of IRF4 after exercise is a systemic effect.

In addition, hindlimb suspension (HLS) of rodents by the tail is a well‐established approach to create a ground‐based model of microgravity and musculoskeletal disuse that mimics many of the physiological changes associated with space flight, prolonged bed rest, as well as with extremely nonexercising.^[^ ^16^, ^17^ ^]^ WT mice were randomly separated into three groups (control/ nonsuspension, unload/ 1 week suspension, reload/reloaded for 7 days after 7 days HLS). In comparison to the controls, the weight of gastrocnemius (GAS) and soleus (SOL) muscle were significantly decreased in unload group and recovered in reload group (Figure S1c,d, Supporting Information). Irf4 messenger ribonucleic acid (mRNA) level was decreased in skeletal muscle from HLS mice and was back to normal after 7 days reload (Figure 1c; Figure S1e, Supporting Information). These data suggest that IRF4 in the skeletal muscle is correlated with physical activity.

### 2.2 Mice Loss of IRF4 in Skeletal Muscle Shows Normal Body Weight and Insulin Sensitivity on Chow Diet

**Figure 2:**
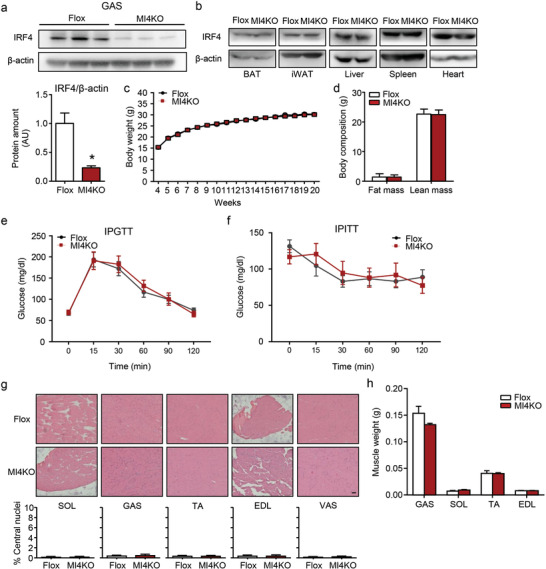
Loss of IRF4 in skeletal muscle shows normal systemic metabolism. a,b) Western blot analysis of the expression of IRF4 in tissues of MI4KO and flox mice. Protein amount was quantified using ImageJ (*n* = 3, *p < 0.05). c) The body weight of male MI4KO and flox mice on chow diet (*n* = 8−10, *p < 0.05). d) The body composition of male MI4KO and flox mice on the age of 20 weeks (*n* = 8−10). e,f) The glucose tolerance test and insulin tolerance test in male MI4KO and flox mice on chow diet at the age of 9 and 10 weeks, respectively (*n* = 8). g) H&E staining of SOL, GAS, TA, EDL, and VAS of male MI4KO and flox mice (scale bars, 50 µm). The percentage of central nuclei was measured by manual counting (*n* = 3). h) The muscle weight of MI4KO and flox mice (*n* = 5). All results are expressed as means ± SEM. SOL, soleus; GAS, gastrocnemius; TA, tibialis anterior; EDL, extensor digitorum longus; VAS, vastus.

We next developed a system by crossing myosin light polypeptide 1 (Myl1)‐cre,^[^ ^18^ ^]^ which is skeletal‐muscle selective, with IRF4 flox mice (Figure S2a, Supporting Information) to target the IRF4 gene and generate skeletal muscle‐specific‐null alleles. The resulting Irf4^flox/flox^;Cre+ animals had remarkably little IRF4 protein left in the skeletal muscle while other tissues, such as fat, heart, and spleen, showed no change (**Figure** **2**a,b). Since muscle tissue contains endothelial cells, Schwann cells associated with axons, satellite cells, adipocytes, and fibroblasts, in addition to skeletal muscle fibers, not all nuclei in skeletal muscle tissue are contained within muscle cells. Indeed, histological studies indicate that ≈45% of the nuclei within skeletal muscle tissue are contained within muscle fibers.^[^ ^19^ ^]^ Therefore, we reasoned that selective and efficient deletion of floxed Irf4 sequences in nuclei from skeletal muscle depot would result in ≈50% deletion of floxed Irf4 sequences in RNA isolated from skeletal muscle tissue (Figure S2b, Supporting Information).

Metabolic phenotypes of MI4KO mice were first calculated. On a regular chow diet, both male and female MI4KO mice displayed no body weight or body composition difference with the control littermates (Figure 2c,d; Figure S2c, Supporting Information). Skeletal muscle is a major tissue involved in glucose metabolism. We then performed the insulin tolerance test and glucose tolerance test (ITT and GTT). These mice have normal GTT and ITT relative to control mice on chow diet in both genders (Figure 2e,f; Figure S2d,e, Supporting Information). We also examined skeletal muscle by microscopy to see whether the absence of IRF4 had any gross morphological consequences. Examination of skeletal muscle sections from the MI4KO mice by hematoxylin and eosin (H&E) staining did not reveal any abnormalities as compared with flox littermates (Figure 2g). There were no significant differences in muscle weights between MI4KO and flox mice (Figure 2h).

### 2.3 Mice Lacking IRF4 in Skeletal Muscle Have Increased Exercise Capacity

**Figure 3:**
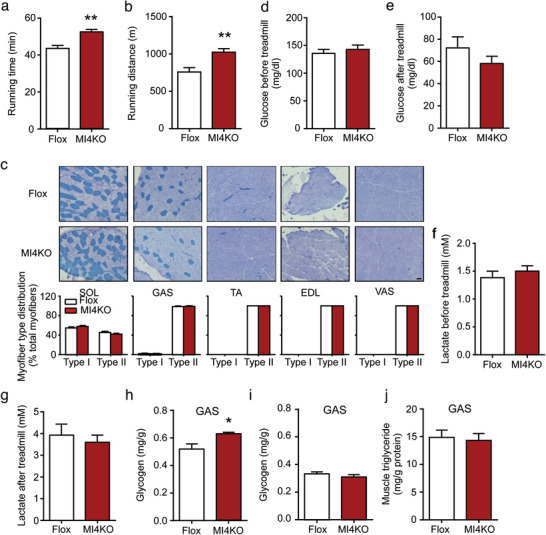
Ablation of IRF4 in skeletal muscle increases the exercise capacity. a,b) The running time and distance of male MI4KO and flox mice (*n* = 10–11, **p < 0.01). c) ATPase staining of SOL, GAS, TA, EDL, and VAS of male MI4KO and flox mice (scale bars, 50 µm). Myofiber type distribution was quantified by manual counting (*n* = 3). d,e) The glucose level of male MI4KO and flox mice before and after running exhaustion (*n* = 10–11). f,g) The lactate level of male MI4KO and flox mice before and after running exhaustion (*n* = 7). h) The glycogen level of GAS in male MI4KO and flox mice before exercise (*n* = 6, *p < 0.05). i) The glycogen level of GAS in male MI4KO and flox mice after exhausting exercise (*n* = 4). j) The muscle triglyceride level in male MI4KO and flox mice (*n* = 6). All results are expressed as means ± SEM.

To test whether IRF4 knockout would affect exercise capacity, we subjected MI4KO and control mice to a high‐intensity treadmill regimen (Figure S3a, Supporting Information). Surprisingly, MI4KO male mice demonstrated increased exercise capacity compared to control mice (**Figure** **3**a,b). Grip strength, however, was unaffected (Figure S3b, Supporting Information). Different muscle fiber types were reported to contribute to exercise capacity.^[^ ^20^, ^21^, ^22^ ^]^ However, that was not the case in our study, as the skeletal muscle displayed a normal distribution of Type I and Type II fibers (Figure 3c). And there were no differences in muscle‐fiber‐type genes (Figure S3c, Supporting Information). We next measured blood glucose levels before and after running to confirm that all mice ran to their limit of fatigue and to test whether differences in blood glucose levels might have affected exercise capacity. Blood glucose levels declined with exercise in both groups, with no significant difference in pre‐ or postexercise levels between the groups (Figure 3d,e). Lactate formation causes acidosis, and muscular fatigue^[^ ^23^, ^24^ ^]^ is present in the periodic literature and in biochemistry, physiology, and exercise physiology textbooks. Recent studies highlight lactate as a biomarker of fatigue rather than as a direct cause.^[^ ^25^, ^26^, ^27^ ^]^ Serum lactate levels were absolutely elevated in both groups after exercise but no significant differences between two groups (Figure 3f,g). Since glycogen depletion can contribute to exercise fatigue (“glycogen shunt” hypothesis),^[^ ^28^ ^]^ we assessed baseline glycogen levels in muscles of both groups and found that MI4KO mice had more glycogen content in skeletal muscle than flox mice before exercise (Figure 3h; Figure S3d,e, Supporting Information). However, the glycogen content in muscle of MI4KO and flox mice was not significantly different after exhausting exercise (Figure 3i), indicating that the increased exercise capacity of MI4KO mice is resulted from increased glycogen content before exercise. The skeletal muscle triglyceride content did not show any differences between the two groups (Figure 3j; Figure S3f, Supporting Information).

### 2.4 Targeted Expression of IRF4 Causes Reduced Exercise Capacity

**Figure 4:**
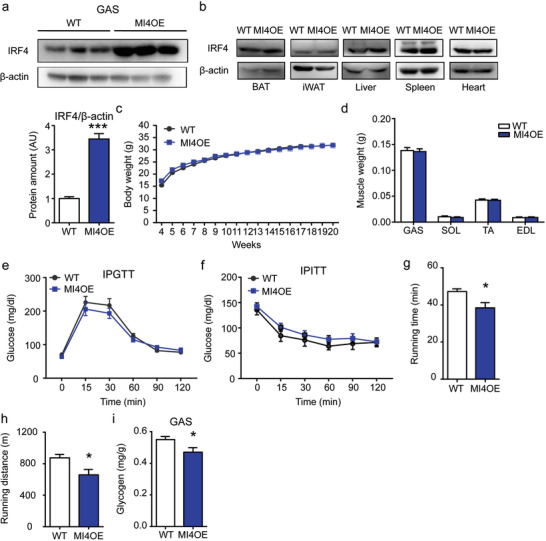
Muscle‐specific IRF4 overexpression mice display reduced exercise capacity. a,b) Western blot analysis of the expression of IRF4 in tissues of MI4OE and WT mice. Protein amount was quantified using ImageJ (*n* = 3, *p < 0.05). c) The body weight of male MI4OE and WT mice on chow diet (*n* = 8–10). d) The muscle weight of MI4OE and WT mice (*n* = 5). e,f) The glucose tolerance test and insulin tolerance test in male MI4OE and WT mice on chow diet at the age of 9 and 10 weeks, respectively (*n* = 8). g,h) The running time and distance of male MI4OE and WT mice (*n* = 8–12, *p < 0.05). i) The glycogen level of GAS in male MI4OE and WT mice (*n* = 8–9, *p < 0.05). All results are expressed as means ± SEM.

We next tested whether overexpression of IRF4 in skeletal muscle (muscle‐specific IRF4 overexpression (MI4OE)) would yield opposing effects to the knockout. By crossing the Myl1‐cre with Rosa26‐LSL‐IRF4 mice^[^ ^29^ ^]^ (Figure S4a, Supporting Information), skeletal muscle Irf4 mRNA was increased more than 100‐fold, but protein only increased by ≈3–5 fold (**Figure** **4**a; Figure S4b, Supporting Information). No overexpression was seen in other tissues (Figure 4b). MI4OE mice showed no significant difference of body weight and muscle weights on chow diet (Figure 4c,d; Figure S4c, Supporting Information). In addition, we addressed the effect of IRF4 overexpression on glucose homeostasis in skeletal muscle. We found that MI4OE mice had normal glucose and insulin tolerance test relative to control mice (Figure 4e,f; Figure S4d,e, Supporting Information).

We also assessed whether overexpression of IRF4 could affect the running ability in mice. MI4OE mice displayed reduced exercise capacity, with lower glycogen levels in skeletal muscle before running (Figure 4g–i). Grip strength was similar in both groups (Figure S4f, Supporting Information).

### 2.5 IRF4 Regulates Glycogen Synthesis Potentially through PTG in Skeletal Muscle

**Figure 5:**
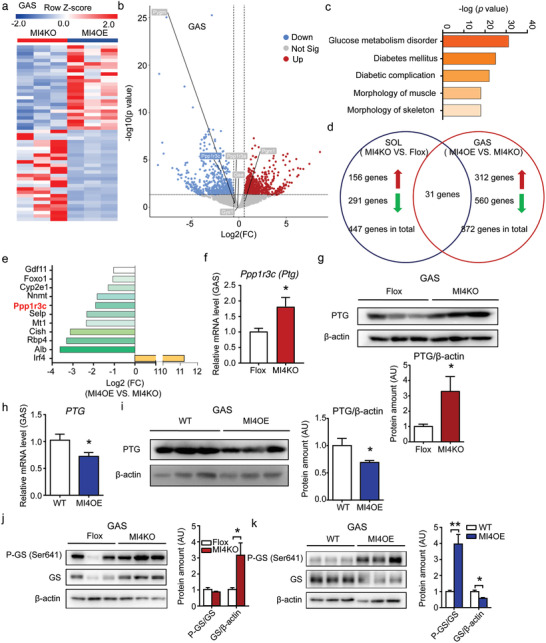
RNA‐seq of skeletal muscle from MI4KO and MI4OE mice. a) Heat map of differentially expressed genes in GAS of MI4KO and MI4OE mice (*n* = 3). b) Volcano plot of differentially expressed genes in GAS of MI4KO and MI4OE mice (Down: *p* < 0.05 and log FC <−0.58; Up: *p* < 0.05 and log FC > 0.58). Genes that relative to glycogen metabolism were labeled. c) The Ingenuity Pathway Analysis (IPA) of RNA‐seq on GAS. d) The comparison of genes that changed in the RNA‐seq of SOL or GAS (Down: *p* < 0.05 and log FC <−0.58; Up: *p* < 0.05 and log FC > 0.58). e) Top ten genes changed in both SOL and GAS. f) qPCR analysis of Ppp1r3c (Ptg) expression in skeletal muscle of MI4KO and flox mice (*n* = 4, *p < 0.05). g) Western blot analysis of the expression of PTG in skeletal muscle of MI4KO and flox mice. Protein amount was quantified using ImageJ (*n* = 6, *p < 0.05). h) qPCR analysis of Ptg expression in skeletal muscle of MI4OE and WT mice (*n* = 6, *p < 0.05). i) Western blot analysis of the expression of PTG in skeletal muscle of MI4OE and WT mice. Protein amount was quantified using ImageJ (*n* = 6, *p < 0.05). j) Western blot analysis of the expression of phospho‐glycogen synthase (P‐GS [Ser641]) and total GS in GAS of MI4KO and flox mice. Protein amount was quantified using ImageJ (*n* = 3, *p < 0.05). k) Western blot analysis of the expression of P‐GS (Ser641) and GS in GAS of MI4OE and WT mice. Protein amount was quantified using ImageJ (*n* = 3, *p < 0.05, **p < 0.01). All results are expressed as means ± SEM.

To further investigate the molecular changes in skeletal muscle induced by IRF4 knockout or overexpression, we performed two independent RNA‐seq assays. One is on SOL muscle from MI4KO and flox mice, and results were normalized to the flox mice; this indicated 447 genes are different corresponding to a |fold‐change| of ≥1.5 and a *p*‐value of <0.05 (**Figure** **5**d; Figure S5a and Table S1, Supporting Information). The other is on GAS muscle from MI4KO and MI4OE mice, and results were normalized to the MI4KO mice; this revealed 872 differentially regulated genes corresponding to a |fold‐change| of ≥1.5 and a *p*‐value of <0.05 (Figure 5a,d; Table S2, Supporting Information). Among the differentially regulated genes in GAS, there were 312 upregulated genes and 560 downregulated genes in overexpressed IRF4 muscle compared to IRF4 knockout muscle tissue (Figure 5d). The Ingenuity Pathway Analysis (IPA) of GAS data showed that most significant differentially expressed genes were related to glucose metabolism disorders, and other pathways involved in diseases and functions associated with metabolism (Figure 5c). Interestingly, when compared changed genes in both SOL and GAS analyses, 31 genes were overlapped (Table S3, Supporting Information). Ppp1r3c (PTG) is not only on the top ten overlapped altered genes list, but also is the only gene related to glycogen synthesis (Figure 5b,d,e; Figure S5b, Supporting Information). PTG is known to bring PP1 and GS into close proximity for the regulation of glycogen synthesis (Figure S5c, Supporting Information). The other muscle‐specific glycogen‐targeting subunit, Ppp1r3a, and glycogen metabolism related genes were not altered in the two groups (Figure 5b; Figure S5b,d, Supporting Information). The Ptg mRNA was nearly 2‐fold increased (Figure 5f) and protein level was about 3‐fold higher in muscle of MI4KO mice compared to flox mice (Figure 5g). Conversely, Ptg mRNA and protein levels were reduced when IRF4 was overexpressed in skeletal muscle (Figure 5h,i). PTG is a critical regulator of PP1, a phosphatase of glycogen synthase. Therefore, we further explored the phosphorylation of GS in the mice. MI4KO mice had a trend toward lower phosphorylation of GS in GAS compared to flox mice (Figure 5j). MI4OE mice had a higher phosphorylation of GS in GAS compared to control mice (Figure 5k) prior to running. Besides these, we found that the total level of GS was changed, increased in skeletal muscle with IRF4 ablation, and decreased in muscle with IRF4 overexpression. These results are consistent with the notion that PTG promotes the dephosphorylation of GS and increases the activity of GS.^[^ ^30^, ^31^, ^32^, ^33^, ^34^ ^]^

Muscle glycogen concentration was reported to be significantly higher in the freely hanging limbs from head‐down suspension rats.^[^ ^35^ ^]^ In this study, we found downregulation of Irf4 (Figure 1c) and upregulation of Ptg expression in hanging limbs (Figure S5e, Supporting Information), which may contribute to the increased glycogen content. Concomitant with this, PTG was decreased after acute exercise (Figure S5f,g, Supporting Information), while IRF4 expression was increased (Figure 1b) and glycogen was decreased. Taken together in the context of IRF4 ablation, these results provide evidence that regulation of PTG expression may be directly responsible for the increased glycogen levels observed in our studies.

### 2.6 PTG Is Required for the Glycogen Induction in IRF4‐Depleted Muscle

**Figure 6:**
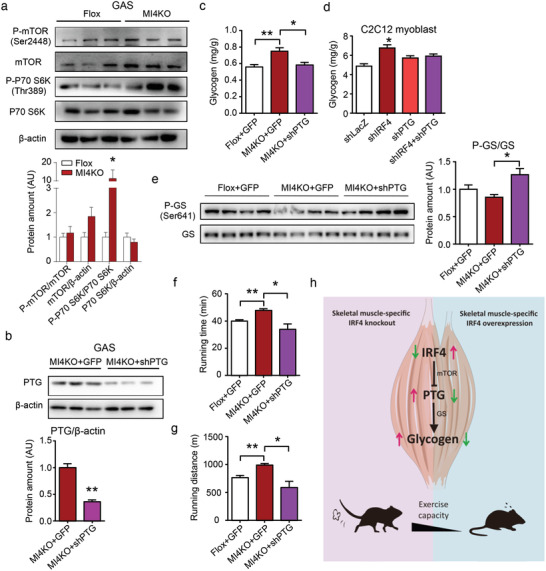
PTG mediates the effect of IRF4 on exercise capacity and glycogen synthesis. a) Western blot analysis of mTOR signaling pathway in GAS of MI4KOmice. Protein amount was quantified using ImageJ (*n* = 3–6, *p < 0.05). b) Western blot analysis of the expression of PTG in skeletal muscle of MI4KO mice with or without AAV–shPTG injection. Protein amount was quantified using ImageJ (*n* = 3, **p < 0.01). c) The glycogen level of GAS in male flox and MI4KO mice with or without AAV–shPTG injection (*n* = 6–7, *p < 0.05 and **p < 0.01). d) The glycogen level of C2C12 myoblast (*n* = 4, *p < 0.05). Cells were transfected with pLKO.1‐shLacZ, pLKO.1‐shIRF4, pLKO.1‐shPTG, or pLKO.1‐shIRF4 with pLKO.1‐shPTG. The cellular glycogen level was measured after 72 h of transfection. e) Western blot analysis of the expression of P‐GS (Ser641) and GS in GAS of flox mice and MKO mice with AAV injection. Protein loading was adjusted to GS total level. Protein amount was quantified using ImageJ (*n* = 4, *p < 0.05). f,g) The running time and distance of male flox and MI4KO mice with or without AAV–shPTG injection (*n* = 6, *p < 0.05 and **p < 0.01). h) A schematic model showing that IRF4 regulates exercise capacity in skeletal muscle. All results are expressed as means ± SEM.

IRF4 has defined roles in transcriptional regulation and binds to interferon‐stimulated response elements (ISRE). Next, we searched ISRE on the promoter of PTG. However, there was no ISRE on PTG promoter, suggesting that IRF4 may indirectly regulate PTG expression. Mammalian target of rapamycin (mTOR)/sterol regulatory element‐binding protein 1 (SREBP1) signaling pathway was reported to regulate PTG expression.^[^ ^10^ ^]^ We thus assessed phosphorylation of mTOR and its targeted gene p70S6K. mTOR signaling was induced in IRF4 disrupted muscle (**Figure** **6**a).

To further test whether PTG mediates the effect of muscle IRF4 loss on glycogen, we carried out a PTG loss‐of‐function approach in vivo. We forced knockdown of PTG directly in the GAS muscle of mice using adeno‐associated viruses (AAV) delivery. Three small hairpin ribonucleic acids (shRNAs) (Table S4, Supporting Information) to target PTG were designed using the BLOCK‐iT RNAi Designer. Ptg expression was 50% knocked down by two of them (Figure S6a, Supporting Information). We thus chose NO.2 to confirm its effect in protein level in cells (Figure S6b, Supporting Information) and then generate AAV. AAV was injected once, and experiments were carried out after 4 weeks recovery. There were no differences in GAS weight between AAV–shPTG and AAV–green fluorescent protein (GFP) injection mice (Figure S6c, Supporting Information). PTG protein was decreased in GAS from AAV–shPTG injected mice compared to that from control mice (Figure 6b). Glycogen content in GAS was back to normal when treated MI4KO mice with AAV–shPTG (Figure 6c). C2C12 cell experiment also showed the same trend of glycogen content (Figure 6d). Additionally, the phosphorylation of GS in GAS of MI4KO mice with AAV‐shPTG was increased compared with MI4KO mice with AAV–GFP (Figure 6e). Even only GAS muscle was injected with AAV–shPTG, it was sufficient to block IRF4 effects on exercise capacity (Figure 6f,g). Our data thus strongly suggest a model in which IRF4 inhibits PTG expression and then regulates glycogen synthesis in skeletal muscle, eventually manipulates exercise capacity (Figure 6h, Work model).

## 3 Discussion

In summary, we have discovered a novel role of IRF4 in skeletal muscle which affects physical activity. IRF4 expression is increased in skeletal muscle of mice (both nontrained and trained) after exercise, whereas in HLS mice it is decreased. Mice lacking IRF4 in the skeletal muscle display a normal metabolic phenotype but induce exercise capacity. Moreover, overexpression of IRF4 shows opposite effects. Metabolically, PTG is required for this effect in skeletal muscle.

For many years, people have been trying to find ways to run for a long time and fast, with very limited improvements. Skeletal muscle glycogen is thought to be critical for exercise capacity.^[^ ^36^ ^]^ Glycogen synthesis and glucose uptake are closely related, since GS and facilitated glucose transporter 4 (GLUT4) are co‐regulated by Akt.^[^ ^37^, ^38^ ^]^ Additionally, adenosine monophosphate‐activated protein kinase (AMPK) activation promotes glucose uptake via facilitated GLUT4 during exercise and increases glycogen synthesis in skeletal muscle.^[^ ^39^, ^40^ ^]^ There are many factors that can alter glucose entry into muscle, including inflammatory/bacterial factors that can impair insulin action and glucose transporter‐mediated uptake of glucose. Klip group investigated that nucleotide oligomerization domain (NOD) proteins contribute to glucose uptake in adipose tissue, liver, and muscle cells.^[^ ^41^, ^42^ ^]^ Schertzer group indicated that IRF4 was responsible for NOD2‐induced insulin sensitizing and anti‐inflammatory effects during obesity and endotoxemia.^[^ ^43^ ^]^ In our study, we did not notice any changes in inflammatory genes changed for IRF4 ablation muscle based on RNA‐seq data. We demonstrated an IRF4‐mediated feedback signaling pathway between exercise and exercise‐induced exhaustion. When running, IRF4 was induced and then inhibited PTG‐associated glycogen synthesis, which eventually led to glycogen depletion and causing exhaustion. The glycogen contents in skeletal muscle was higher in MI4KO mice than in flox mice prior to exercise. However, after running until they were exhausted, the levels of glycogen were similar in both models. In addition, GS protein level was higher and phosphorylation level had a trend to be lower in IRF4 ablation muscle. These results suggest that glycogen synthesis and re‐synthesis are critical for IRF4 to suppress exercise capacity. We previously found that brown adipose‐tissue‐specific IRF4 knockout (BATI4KO) mice exhibit decreased exercise capacity. Furthermore, loss of IRF4 in BAT increases myostatin expression, which plays a role in BAT and skeletal muscle crosstalk.^[^ ^44^ ^]^ However, the MI4KO mice showed increased exercise capacity, and there is no altered myostatin expression in IRF4 ablation skeletal muscle compared to control. This suggested that IRF4 regulates exercise capacity in a tissue‐specific manner. Another study reported a role of IRF4 in the regulation of pathological cardiac hypertrophy, which mediated by cyclic adenosine monophosphate (cAMP) response element‐binding protein (CREB).^[^ ^45^ ^]^ Moreover, CREB/cAMP in skeletal muscle can regulate exercise performance and glycogen content.^[^ ^46^ ^]^ These studies suggested that IRF4 may regulate glycogen through CREB. However, we did not see any CREB‐responsive genes expression altered, or muscle hypertrophy (shown in activation of the CREB study) in MI4KO mice compared to controls. Other than that, increased PTG expression and glycogen content in IRF4 deleted skeletal muscle contribute to improved exercise capacity.

Moreover, the expression of IRF4 was upregulated not only in skeletal muscle but also in adipose tissue and heart after exercise, indicating that exercise exerts a systemic effect on IRF4 expression. How does exercise regulate IRF4 expression? One piece of our previous work focused on the role of IRF4 in brown fat and found PGC‐1*α* may regulate IRF4 expression when exposed to cold stress.^[^ ^15^ ^]^ Another work from Rosen group suggested that Foxo1 can mediate insulin signaling on IRF4 expression.^[^ ^13^ ^]^ PGC‐1*α* and Foxo1 are both very important factors to regulate energy homeostasis in metabolic tissues and they are responsive to exercise.^[^ ^47^, ^48^, ^49^, ^50^ ^]^ PGC‐1*α* and Foxo1 could be involved in regulating exercise‐induced IRF4 expression in different tissues. Except for those, endocrine response, stress, or nervous system may be considered as well. Further studies need to be done.

In order to illustrate what kind of gene sets altered in skeletal muscle, we performed two RNA‐seq assays on SOL and GAS, respectively. Both assays showed changes in hundreds of genes, buy only 31 genes out of the 447 in SOL and 872 in GAS overlapped. This may be that SOL and GAS have different proportions of fiber types, which leads to different function and metabolism. SOL is predominantly type I fiber, whereas GAS is a type I and type II fiber mixed muscle.^[^ ^51^ ^]^ Type I fiber is responsible for slow twitch, whereas type II fiber is responsible for fast twitch. Thus, they have significant differences in function and metabolism, hence the reason why only 31 genes overlapped. Among the 31 genes, one of the significantly upregulated genes was PTG, which regulates glucose metabolism. Other genes, such as Foxo1 and Nnmt, are involved in energy metabolism as well. However, whether they are responsible for IRF4‐induced activity is not clear yet, and further studies need to be done. IPA analysis indicates that loss of IRF4 in skeletal muscle may contribute to glucose metabolism disorder. However, neither MI4KO nor MI4OE mice showed any body weight or insulin sensitivity differences on normal chow diet. To investigate whether IRF4 in skeletal muscle has a role in regulating systemic energy homeostasis, further experiments need to be done, thus, when using high‐fat diet on mice. Those studies are still ongoing.

PTG, a scaffolding protein that targets protein PP1 to glycogen, is reported to play a critical role in glycogen synthesis.^[^ ^8^, ^10^, ^52^ ^]^ Study showed that heterozygous PTG deletion did not lead to impaired glycogen synthesis and reduced glycogen levels in GAS.^[^ ^52^ ^]^ In our study, we also found that knockdown of PTG in C2C12 myoblast did not significantly change the content of glycogen. However, the glycogen level in GAS of MI4KO mice with AAV–shPTG injection was significantly reduced. The reason of this inconsistency could be that PTG does not affect the basal level of glycogen, whereas it regulates glycogen contents induced by IRF4 ablation in skeletal muscle. Despite lack of studies on PTG's role in skeletal muscle, disruption of striated muscle Ppp1r3a leads to increased body weight and fat deposition, whereas PTG showed compensatory stimulation by insulin in the absence of Ppp1r3a.^[^ ^53^ ^]^ PTG expression is increased in the absence of IRF4 and decreased when IRF4 is overexpressed in skeletal muscle. Knockdown of PTG in vivo can block the effects of IRF4 deletion. As a result, we conclude that PTG is a potential mediator that regulates IRF4's function in glycogen synthesis in skeletal muscle. Then the coming question is how IRF4 regulates PTG expression. As a transcriptional factor, IRF4 can regulate lots of genes in immune (IL‐4,^[^ ^54^ ^]^ Bcl‐6, CD23, and IL‐1*β* ^[^ ^55^ ^]^) and in metabolism (hormone‐sensitive lipase (HSL), adipose triglyceride lipase (ATGL),^[^ ^13^ ^]^ and uncoupling protein 1 (UCP1)^[^ ^15^ ^]^). However, there is no IRF4 binding elements on the PTG promoter. Hypoxia‐inducible factor 1 (HIF1)^[^ ^56^ ^]^ and forkhead protein FOXA2^[^ ^57^ ^]^ were reported to transcriptionally regulate PTG. We did not see any HIF1 expression differences in skeletal muscle of MI4KO compared to flox mice. Additionally, FOXA2 was rarely expressed in muscle. Conversely, mTOR and SREBP pathways were reported to regulate PTG expression in liver.^[^ ^10^ ^]^ We have reported that IRF4 can regulate mTOR signaling in BAT.^[^ ^15^ ^]^ Here, mTOR pathway was also induced in IRF4‐deleted muscle. We therefore presumed that IRF4 may regulate PTG through mTOR pathway, not directly binding at transcriptional level.

Given numerous benefits of exercise in preventing metabolic diseases, identification of druggable targets that mimic or boost the molecular cascade of endurance training has been a long‐standing, but elusive, medical goal.^[^ ^58^ ^]^ Our study identifies IRF4 as the signaling node that regulates metabolic adaptation to extracellular stimuli; this is supported by our findings that IRF4 levels in skeletal muscle were increased by exercise, and that the genetic IRF4 KO mouse model facilitated a reprogramming of glycogen synthesis. MI4KO mice showed endurance exercise capacity without previous training. Thus, we propose that IRF4 may play an important role in manipulating exercise fatigue.

## 4 Experimental Section

##### Animals

B6.129S1‐Irf4^tm1Rdf^/J mice (IMSR_JAX:0 09380) and Myl1^tm1(cre)Sjb^/J mice (IMSR_JAX:02 4713) were purchased from Jackson Laboratory. Rosa26‐LSL‐IRF4 mice were generated as previously reported.^[^ ^17^ ^]^ Mice were maintained under a 12 h light/12 h dark cycle at constant temperature (23 °C) with free access to food and water. *Irf4* ^flox/flox^ mice were first crossed to Myl1‐Cre mice. The MI4KO mice were obtained by mating F1*Irf4* ^flox/+^;Cre^+^ mice to littermate *Irf4* ^flox/+^;Cre^−^ mice. The MI4OE mice were obtained by crossing Myl1‐Cre mice with Rosa26‐LSL‐Irf4 mice. All animal studies were approved by the Institutional Animal Care and Use Committee of UCLA.

##### Exercise Protocol 1: Long‐Term Training Exercise Followed by an Acute Exercise

8 week male C57BL/6J WT mice were individually housed in cages with free access to a running wheel (free wheel running). Mice were exercised for 30 days and then randomly divided into the sedentary and exercise groups (one‐bout treadmill exercise). For treadmill exercise, mice were acclimated to the treadmill by running for 10 min at 10 m min^−1^ for 2 days prior to experimentation. On the third day, mice run the treadmill at 12 m min^−1^ with 5° incline for 90 min.

##### Exercise Protocol 2: Acute Endurance Treadmill Exercise

8 week male C57BL/6J WT mice were randomly divided into the sedentary, 0 h post exercise, and 3 h post exercise groups.^[^ ^59^ ^]^ Mice in the sedentary group were fasted for 6 h prior to tissue harvest. Mice in the 0 h post exercise and 3 h post exercise groups were fasted for 3 h prior to exercise. Mice were acclimated to the treadmill for 2 days and run the treadmill on the third day as described in the “Exercise Protocol 1: Long‐Term Training Exercise Followed by an Acute Exercise” Section. Mice in the 0 h post exercise group were sacrificed immediately post exercise. Mice in the 3 h post exercise group were sacrificed 3 h post exercise.

##### Exercise Capacity Measurement

Mice at the age of 8 weeks were required an acclimation period of 2 days to the treadmill before the exercise test. For adaptive exercise, the treadmill was set at a 20° incline and began with a 5 min 0 m min^−1^ acclimation period, followed by 10 m min^−1^ for 10 min and 14 m min^−1^ for another 10 min. For exercise test, the treadmill was set at a 10° incline, began at a speed of 10 m min^−1^ for 5 min, and increased by 2 m min^−1^ every 5 min until the mice were exhausted (mice spent more than 5 s on the electric shocker without resuming running).

##### HLS Model

The HLS method was performed as previous described.^[^ ^60^ ^]^ Briefly, 10 week male mice were HLS for 7 days. The reloaded mice were suspension for 7 days and then reloaded for another 7 days. The weight of skeletal muscle was measured after the mice were sacrificed.

##### Body Composition Measurement

The body composition of mice was noninvasively measured using the Minispec mq10 NMR Analyzer (Bruker) according to the manufacturer's instructions.

##### Glucose and Insulin Tolerance Tests

For GTT, mice were fasted overnight before experiment. Glucose (1 g kg^−1^) was administered intraperitoneally (i.p.), and blood glucose levels were measured at 0, 15, 30, 60, and 120 min after injection by using a blood glucose meter (Bayer). For ITT, mice were fasted for 6 h before experiment. Insulin (0.7 U kg^−1^) was administered i.p., and blood glucose was measured at 0, 15, 30, 60, and 120 min after injection.

##### Histology

For H&E staining, skeletal muscles of male mice were harvested and fixed in 10% formalin (Sigma–Aldrich) and embedded in paraffin wax. 5 µm sections were cut and stained with hematoxylin and eosin using a standard protocol. For ATPase staining, skeletal muscles of male mice were harvested and frozen in embedding medium containing a 3:1 mixture of tissue freezing medium and gum tragacanth. ATPase staining was performed as described previously.^[^ ^61^, ^62^ ^]^ Briefly, sections were cut and preincubated for 15 min at pH 4.5 and incubated for 45 min at pH 9.4 for the standard ATPase reaction.

##### Glucose and Lactate Measurement

The blood lactate and glucose of mice were measured before and after treadmill by using a blood glucose meter (Bayer) and a blood lactate meter (Nova Biomedical), respectively.

##### Glycogen Extraction and Measurement

Muscle tissue (10 mg) was rapidly homogenized with 200 µL double distilled water (ddH_2_O) for 10 min on ice. Then, the homogenates were boiled for 10 min to inactivate enzymes. After that, the homogenates were centrifuged at 18 000 rpm for 10 min at 4 °C. The supernatant was collected and measured via using Glycogen Assay Kit II (Abcam) according to the manufacturer's instructions.

##### Triglyceride Extract and Measurement

The lipid of skeletal muscle was extracted by using the chloroform/methanol method as previously described.^[^ ^63^ ^]^ Briefly, skeletal muscle was homogenized in phosphate‐buffered saline (PBS) and then extracted using chloroform/methanol (2:1). After drying, the precipitate was resuspended in ethanol. Total triglyceride level in skeletal muscle was measured by using l‐type Triglyceride Assay Kit (FUJIFILM Wako Diagnostics USA) according to the manufacturer's instructions.

##### Protein Extraction and Western Blot Analysis

Proteins were extracted with radioimmunoprecipitation assay (RIPA) buffer (Boston BioProducts) containing protease and phosphatase inhibitors (Thermo Fisher). Protein concentration was quantified using Rapid Gold BCA Protein Assay Kit (Thermo Fisher) according to the manufacturer's instructions. For western blot analysis, 40 µg of lysate was loaded onto sodium dodecyl sulphate‐polyacrylamide gel electrophoresis (SDS‐PAGE) gels, blotted onto polyvinylidenedifluoride (PVDF) membranes (Millipore), and incubated with antibodies. IRF4 (RRID: AB_2 264 940) and PTG (MBS9413679) rabbit polyclonal antibody were purchased from Proteintech and MyBioSource, respectively. Phospho‐glycogen synthase (Ser641) (94 905), glycogen synthase (RRID:AB_2 279 563), *β*‐Actin mouse (RRID: AB_330 288), phospho‐mTOR (Ser2448) (RRID: AB_330 970), mTOR (RRID: AB_330 978), phospho‐p70 S6 kinase (Thr389) (RRID: AB_2 269 803), and p70 S6 kinase (RRID: AB_390 722) antibodies were purchased from Cell Signaling Technology. Protein amount was measured using ImageJ Java 1.6.0_2.4 software.

##### Analysis of Gene Expression by Quantitative Real‐Time PCR

Total RNA was extracted using the TRIzol method (Thermo Fisher) according to the manufacturer's instructions. A total of 1 µg of RNA was converted into complementary deoxyribonucleic acid (cDNA) using High‐Capacity cDNA Reverse Transcription Kit (Thermo Fisher) according to the manufacturer's instructions. Quantitative real‐time PCR (qPCR) was performed with a QuantStudio 6 Flex Real‐Time PCR System (Thermo Fisher) using SYBR Green PCR Master Mix (Life Technologies) according to the manufacturer's instructions. The relative abundance of mRNAs was standardized with Tbp mRNA as the invariant control. Table S4 (Supporting Information) lists the sequence of the primers used in this study.

##### Plasmids’ Construction

The pENTR/U6‐shPTG encoding three different shRNAs targeting mouse PTG was constructed by inserting small hairpin RNA sequence into the pENTR/U6 vector according to the manufacturer's instructions. The sequence of three different shRNAs targeting mouse PTG was listed in Table S4 (Supporting Information).

##### Cell lines, Culture Conditions, and Transfection

C2C12 myoblasts were grown in high glucose Dulbecco's Modified Eagle Medium (DMEM, Gibco) with 10% fetal bovine serum (FBS, Gibco) and 1% penicillin–streptomycin (P/S, Gibco) in a 5% CO_2_ atmosphere at 37 °C. For plasmid transfection, lipofectamine LTX & PLUS was used according to the instructions of manufacturer (Thermo Fisher).

##### AAV–shPTG Production, Purification, and Injection

AAV2/9‐U6 shPTG‐CMV‐GFP was produced and purified by Boston Children's Hospital Viral Core using sequence (shPTG‐2) as shown in Table S4 (Supporting Information). For PTG knockdown experiment in vivo, a dose of 1.3 × 10^11^ genome copy (GC) of AAV–shPTG was injected into the gastrocnemius muscle of MI4KO mice. A dose of 1.3 × 10^11^ GC of AAV–GFP was injected into the gastrocnemius muscle of control groups. After 4 weeks, the exercise capacity was measured. The mice were sacrificed 4 days post exercise.

##### RNA‐seq Library Generation, Sequencing, and Analysis

A total of 14 samples (4 SOL from MI4KO mice and 4 soleus from flox mice; 3 gastrocnemius from MI4KO mice and 3 GAS from MI4OE mice) were used for RNA‐seq. Libraries for RNA‐Seq were generated with Kapa Stranded mRNA. The data were sequenced on HiSeq3000 using a single‐read 50 bp read run. Data quality check was done on Illumina SAV. Demultiplexing was performed with Illumina Bcl2fastq2 v 2.17 program. The reads were mapped to the latest UCSC transcript set using Bowtie2 version 2.1.0,^[^ ^64^ ^]^ and the gene expression level was estimated using RSEM v1.2.15.^[^ ^65^ ^]^ Trimmed mean of *M*‐values (TMM) was used to normalize the gene expression. Differentially expressed genes were identified using the edgeR program,^[^ ^66^ ^]^ as defined by having a fold change of at least 1.5 and a *p*‐value of <0.05. IPA was performed for subsequent pathway analysis.^[^ ^67^ ^]^

##### Statistical Analysis

All data were presented as mean ± standard error of mean (SEM). For two groups, unpaired two‐tailed Student's *t*‐test or nonparametric test was performed for comparison. For more than two groups, one‐way analysis of variance (ANOVA) and posthoc multiple comparison (least significant difference (LSD) method) were performed for intergroup comparisons. *p* < 0.05 was considered statistically significant.

##### Additional Information

Accession codes: The RNA‐seq data have been deposited in Gene Expression Omnibus (GEO) under the accession code. All bioinformatics software used in the study are publicly available.

##### Data Availability

The accession number for the RNA‐Seq data reported in this paper is GEO: GSE136623 and GSE140633. All bioinformatics software used in the study are publicly available.

## Conflict of Interest

The authors declare no conflict of interest.

## Author Contributions

X.Z., T.Y., and R.W. contributed equally to this work. The experimental plan was designed by T.Y., X.Z., R.W., J.Z.L., T.L., and X.K. T.Y., X.Z., Z.Z., S.G., X.W., X.Z., and Y.Z. performed experiments and analyzed data. R.W. and X.Z. did the revised experiment. RNA‐Seq data were analyzed by T.Y., R.T.W., and X.Z. X.K. and X.Z. wrote the manuscript incorporating edits and comments from T.L., J.L., and all other authors.

## Supporting information

Supporting Information

Supplemental Table 1

Supplemental Table 2

## References

[1] C. E. Xirouchaki , S. P. Mangiafico , K. Bate , Z. Ruan , A. M. Huang , B. W. Tedjosiswoyo , B. Lamont , W. Pong , J. Favaloro , A. R. Blair , J. D. Zajac , J. Proietto , S. Andrikopoulos , Mol. Metab. 2016, 5, 221. 26977394 10.1016/j.molmet.2016.01.004 PMC4770268

[2] P. D. Balsom , G. C. Gaitanos , K. Soderlund , B. Ekblom , Acta Physiol. Scand. 1999, 165, 337. 10350228 10.1046/j.1365-201x.1999.00517.x

[3] J. Bergstrom , L. Hermansen , E. Hultman , B. Saltin , Acta Physiol. Scand. 1967, 71, 140. 5584523 10.1111/j.1748-1716.1967.tb03720.x

[4] N. Ortenblad , H. Westerblad , J. Nielsen , J. Physiol. 2013, 591, 4405. 23652590 10.1113/jphysiol.2013.251629 PMC3784189

[5] S. Guo , Y. Huang , Y. Zhang , H. Huang , S. Hong , T. Liu , J. Sport Health Sci. 2020, 9, 53. 31921481 10.1016/j.jshs.2019.07.004 PMC6943779

[6] S. Munro , D. J. Cuthbertson , J. Cunningham , M. Sales , P. T. Cohen , Diabetes 2002, 51, 591. 11872655 10.2337/diabetes.51.3.591

[7] S. Munro , H. Ceulemans , M. Bollen , J. Diplexcito , P. T. Cohen , FEBS J. 2005, 272, 1478. 15752363 10.1111/j.1742-4658.2005.04585.x

[8] J. A. Printen , M. J. Brady , A. R. Saltiel , Science 1997, 275, 1475. 9045612 10.1126/science.275.5305.1475

[9] M. J. Brady , J. A. Printen , C. C. Mastick , A. R. Saltiel , J. Biol. Chem. 1997, 272, 20198. 9242697 10.1074/jbc.272.32.20198

[10] B. Lu , D. Bridges , Y. Yang , K. Fisher , A. Cheng , L. Chang , Z. X. Meng , J. D. Lin , M. Downes , R. T. Yu , C. Liddle , R. M. Evans , A. R. Saltiel , Diabetes 2014, 63, 2935. 24722244 10.2337/db13-1531 PMC4141363

[11] R. Yang , L. Cao , R. Gasa , M. J. Brady , A. D. Sherry , C. B. Newgard , J. Biol. Chem. 2002, 277, 1514. 11600496 10.1074/jbc.M107001200

[12] R. Gasa , P. B. Jensen , H. K. Berman , M. J. Brady , A. A. Depaoli‐Roach , C. B. Newgard , J. Biol. Chem. 2000, 275, 26396. 10862764 10.1074/jbc.M002427200

[13] J. Eguchi , Q. W. Yan , D. E. Schones , M. Kamal , C. H. Hsu , M. Q. Zhang , G. E. Crawford , E. D. Rosen , Cell Metab. 2008, 7, 86. 18177728 10.1016/j.cmet.2007.11.002 PMC2278019

[14] J. Eguchi , X. Kong , M. Tenta , X. Wang , S. Kang , E. D. Rosen , Diabetes 2013, 62, 3394. 23835343 10.2337/db12-1327 PMC3781469

[15] X. Kong , A. Banks , T. Liu , L. Kazak , R. R. Rao , P. Cohen , X. Wang , S. Yu , J. C. Lo , Y. H. Tseng , A. M. Cypess , R. Xue , S. Kleiner , S. Kang , B. M. Spiegelman , E. D. Rosen , Cell 2014, 158, 69. 24995979 10.1016/j.cell.2014.04.049 PMC4116691

[16] E. Morey‐Holton , R. K. Globus , A. Kaplansky , G. Durnova , Adv. Space Biol. Med. 2005, 10, 7. 16101103 10.1016/s1569-2574(05)10002-1

[17] E. R. Morey‐Holton , R. K. Globus , J. Appl. Physiol. 2002, 92, 1367. 11895999 10.1152/japplphysiol.00969.2001

[18] G. W. Bothe , J. A. Haspel , C. L. Smith , H. H. Wiener , S. J. Burden , genesis 2000, 26, 165. 10686620

[19] H. Schmalbruch , U. Hellhammer , The Anat. Rec. 1977, 189, 169. 911042 10.1002/ar.1091890204

[20] J. M. Wilson , J. P. Loenneke , E. Jo , G. J. Wilson , M. C. Zourdos , J. S. Kim , J. Strength Cond. Res. 2012, 26, 1724. 21912291 10.1519/JSC.0b013e318234eb6f

[21] A. Nilsson , E. Bjornson , M. Flockhart , F. J. Larsen , J. Nielsen , Nat. Commun. 2019, 10, 5072. 31699973 10.1038/s41467-019-12934-8 PMC6838197

[22] P. A. Dutchak , S. J. Estill‐Terpack , A. A. Plec , X. Zhao , C. Yang , J. Chen , B. Ko , R. J. Deberardinis , Y. Yu , B. P. Tu , Cell Rep. 2018, 23, 1907. 29768191 10.1016/j.celrep.2018.04.058 PMC6038807

[23] K. Klausen , H. G. Knuttgen , H. V. Forster , Scand. J. Clin. Lab. Invest. 1972, 30, 415. 4639646 10.3109/00365517209080279

[24] J. Karlsson , F. Bonde‐Petersen , J. Henriksson , H. G. Knuttgen , J. Appl. Physiol. 1975, 38, 763. 1126883 10.1152/jappl.1975.38.5.763

[25] S. T. Knuth , H. Dave , J. R. Peters , R. H. Fitts , J. Physiol. 2006, 575, 887. 16809373 10.1113/jphysiol.2006.106732 PMC1995695

[26] O. B. Nielsen , F. De Paoli , K. Overgaard , J. Physiol. 2001, 536, 161. 11579166 10.1111/j.1469-7793.2001.t01-1-00161.x PMC2278832

[27] O. B. Nielsen , K. Overgaard , J. Appl. Physiol. 2006, 101, 367 [author reply 369]. 10.1152/japplphysiol.00181.2006 16782838

[28] R. G. Shulman , D. L. Rothman , Proc. Natl. Acad. Sci. USA 2001, 98, 457. 11209049 10.1073/pnas.98.2.457 PMC14608

[29] X. Kong , K. W. Williams , T. Liu , Methods Mol. Biol. 2017, 1566, 99. 28244044 10.1007/978-1-4939-6820-6_10 PMC5572801

[30] A. M. Danos , S. Osmanovic , M. J. Brady , J. Biol. Chem. 2009, 284, 19544. 19487702 10.1074/jbc.M109.015073 PMC2740580

[31] M. Montori‐Grau , M. Guitart , C. Garcia‐Martinez , A. Orozco , A. M. Gomez‐Foix , BMC Biochem. 2011, 12, 57. 22054094 10.1186/1471-2091-12-57 PMC3240831

[32] C. Lerin , E. Montell , H. K. Berman , C. B. Newgard , A. M. Gomez‐Foix , J. Biol. Chem. 2000, 275, 39991. 10998419 10.1074/jbc.M006251200

[33] C. B. Newgard , M. J. Brady , R. M. O'doherty , A. R. Saltiel , Diabetes 2000, 49, 1967. 11117996 10.2337/diabetes.49.12.1967

[34] I. Lopez‐Soldado , D. Zafra , J. Duran , A. Adrover , J. Calbo , J. J. Guinovart , Diabetes 2015, 64, 796. 25277398 10.2337/db14-0728

[35] C. S. Stump , J. M. Overton , C. M. Tipton , J. Appl. Physiol. 1990, 68, 627. 2318773 10.1152/jappl.1990.68.2.627

[36] P. J. Roach , A. A. Depaoli‐Roach , T. D. Hurley , V. S. Tagliabracci , Biochem. J. 2012, 441, 763. 22248338 10.1042/BJ20111416 PMC4945249

[37] G. I. Welsh , I. Hers , D. C. Berwick , G. Dell , M. Wherlock , R. Birkin , S. Leney , J. M. Tavare , Biochem. Soc. Trans. 2005, 33, 346. 15787603 10.1042/BST0330346

[38] Q. Li , Q. Zhao , J. Zhang , L. Zhou , W. Zhang , B. Chua , Y. Chen , L. Xu , P. Li , Cell Rep. 2019, 28, 3406. 31553910 10.1016/j.celrep.2019.08.066

[39] J. R. Hingst , L. Bruhn , M. B. Hansen , M. F. Rosschou , J. B. Birk , J. Fentz , M. Foretz , B. Viollet , K. Sakamoto , N. J. Faergeman , J. F. Havelund , B. L. Parker , D. E. James , B. Kiens , E. A. Richter , J. Jensen , J. F. P. Wojtaszewski , Mol. Metab. 2018, 16, 24. 30093357 10.1016/j.molmet.2018.07.001 PMC6158101

[40] E. A. Richter , M. Hargreaves , Physiol. Rev. 2013, 93, 993. 23899560 10.1152/physrev.00038.2012

[41] A. K. Tamrakar , J. D. Schertzer , T. T. Chiu , K. P. Foley , P. J. Bilan , D. J. Philpott , A. Klip , Endocrinology 2010, 151, 5624. 20926588 10.1210/en.2010-0437

[42] J. D. Schertzer , A. K. Tamrakar , J. G. Magalhaes , S. Pereira , P. J. Bilan , M. D. Fullerton , Z. Liu , G. R. Steinberg , A. Giacca , D. J. Philpott , A. Klip , Diabetes 2011, 60, 2206. 21715553 10.2337/db11-0004 PMC3161332

[43] J. F. Cavallari , M. D. Fullerton , B. M. Duggan , K. P. Foley , E. Denou , B. K. Smith , E. M. Desjardins , B. D. Henriksbo , K. J. Kim , B. R. Tuinema , J. C. Stearns , D. Prescott , P. Rosenstiel , B. K. Coombes , G. R. Steinberg , J. D. Schertzer , Cell Metab. 2017, 25, 1063. 28434881 10.1016/j.cmet.2017.03.021

[44] X. Kong , T. Yao , P. Zhou , L. Kazak , D. Tenen , A. Lyubetskaya , B. A. Dawes , L. Tsai , B. B. Kahn , B. M. Spiegelman , T. Liu , E. D. Rosen , Cell Metab. 2018, 28, 631. 30078553 10.1016/j.cmet.2018.07.004 PMC6170693

[45] D. S. Jiang , Z. Y. Bian , Y. Zhang , S. M. Zhang , Y. Liu , R. Zhang , Y. Chen , Q. Yang , X. D. Zhang , G. C. Fan , H. Li , Hypertension 2013, 61, 1193. 23589561 10.1161/HYPERTENSIONAHA.111.00614 PMC3734933

[46] N. E. Bruno , K. A. Kelly , R. Hawkins , M. Bramah‐Lawani , A. L. Amelio , J. C. Nwachukwu , K. W. Nettles , M. D. Conkright , EMBO J. 2014, 33, 1027. 24674967 10.1002/embj.201386145 PMC4193935

[47] D. Constantin‐Teodosiu , D. Constantin , F. Stephens , D. Laithwaite , P. L. Greenhaff , Diabetes 2012, 61, 1017. 22315317 10.2337/db11-0799 PMC3331777

[48] S. B. Jorgensen , J. F. Wojtaszewski , B. Viollet , F. Andreelli , J. B. Birk , Y. Hellsten , P. Schjerling , S. Vaulont , P. D. Neufer , E. A. Richter , H. Pilegaard , FASEB J. 2005, 19, 1146. 15878932 10.1096/fj.04-3144fje

[49] H. Pilegaard , B. Saltin , P. D. Neufer , J. Physiol. 2003, 546, 851. 12563009 10.1113/jphysiol.2002.034850 PMC2342594

[50] A. Safdar , J. P. Little , A. J. Stokl , B. P. Hettinga , M. Akhtar , M. A. Tarnopolsky , J. Biol. Chem. 2011, 286, 10605. 21245132 10.1074/jbc.M110.211466 PMC3060512

[51] S. Schiaffino , C. Reggiani , Physiol. Rev. 2011, 91, 1447. 22013216 10.1152/physrev.00031.2010

[52] S. M. Crosson , A. Khan , J. Printen , J. E. Pessin , A. R. Saltiel , J. Clin. Invest. 2003, 111, 1423. 12727934 10.1172/JCI17975 PMC154451

[53] M. Delibegovic , C. G. Armstrong , L. Dobbie , P. W. Watt , A. J. Smith , P. T. Cohen , Diabetes 2003, 52, 596. 12606498 10.2337/diabetes.52.3.596

[54] S. Gupta , M. Jiang , A. Anthony , A. B. Pernis , J. Exp. Med. 1999, 190, 1837. 10601358 10.1084/jem.190.12.1837 PMC2195723

[55] S. Marecki , M. J. Fenton , J. Interferon Cytokine Res. 2002, 22, 121. 11846983 10.1089/107999002753452737

[56] G. M. Shen , F. L. Zhang , X. L. Liu , J. W. Zhang , FEBS Lett. 2010, 584, 4366. 20888814 10.1016/j.febslet.2010.09.040

[57] A. Cheng , M. Zhang , S. M. Crosson , Z. Q. Bao , A. R. Saltiel , Endocrinology 2006, 147, 3606. 16627590 10.1210/en.2005-1513

[58] X. Luan , X. Tian , H. Zhang , R. Huang , N. Li , P. Chen , R. Wang , J. Sport Health Sci. 2019, 8, 422. 31534817 10.1016/j.jshs.2019.04.002 PMC6742679

[59] T. M. Moore , Z. Zhou , W. Cohn , F. Norheim , A. J. Lin , N. Kalajian , A. R. Strumwasser , K. Cory , K. Whitney , T. Ho , T. Ho , J. L. Lee , D. H. Rucker , O. Shirihai , A. M. Van Der Bliek , J. P. Whitelegge , M. M. Seldin , A. J. Lusis , S. Lee , C. A. Drevon , S. K. Mahata , L. P. Turcotte , A. L. Hevener , Mol. Metab. 2019, 21, 51. 30591411 10.1016/j.molmet.2018.11.012 PMC6407367

[60] J. A. Ferreira , J. M. Crissey , M. Brown , J. Vis. Exp. 2011, 49, pii: 2467. 10.3791/2467 PMC3197292 21445032

[61] N. Liu , S. Bezprozvannaya , J. M. Shelton , M. I. Frisard , M. W. Hulver , R. P. Mcmillan , Y. Wu , K. A. Voelker , R. W. Grange , J. A. Richardson , R. Bassel‐Duby , E. N. Olson , J. Clin. Invest. 2011, 121, 3258. 21737882 10.1172/JCI46267 PMC3148737

[62] M. H. Brooke , K. K. Kaiser , Arch. Neurol. 1970, 23, 369. 4248905 10.1001/archneur.1970.00480280083010

[63] M. Zang , S. Xu , K. A. Maitland‐Toolan , A. Zuccollo , X. Hou , B. Jiang , M. Wierzbicki , T. J. Verbeuren , R. A. Cohen , Diabetes 2006, 55, 2180. 16873680 10.2337/db05-1188

[64] B. Langmead , S. L. Salzberg , Nat. Methods 2012, 9, 357. 22388286 10.1038/nmeth.1923 PMC3322381

[65] B. Li , C. N. Dewey , BMC Bioinformatics 2011, 12, 323. 21816040 10.1186/1471-2105-12-323 PMC3163565

[66] M. D. Robinson , D. J. Mccarthy , G. K. Smyth , Bioinformatics 2010, 26, 139. 19910308 10.1093/bioinformatics/btp616 PMC2796818

[67] A. Kramer , J. Green , J. Pollard Jr. , S. Tugendreich , Bioinformatics 2014, 30, 523. 24336805 10.1093/bioinformatics/btt703 PMC3928520

