# Supplementary material for: IRF4 in Skeletal Muscle Regulates Exercise Capacity via PTG/Glycogen Pathway
Source: Adv Sci (Weinh). 2020 Aug 1;7(19):2001502. doi: 10.1002/advs.202001502 (PMC7539189; doi:10.1002/advs.202001502)
Supplement: Supplementary file 1 — Supporting Information [file ADVS-7-2001502-s001.pdf]

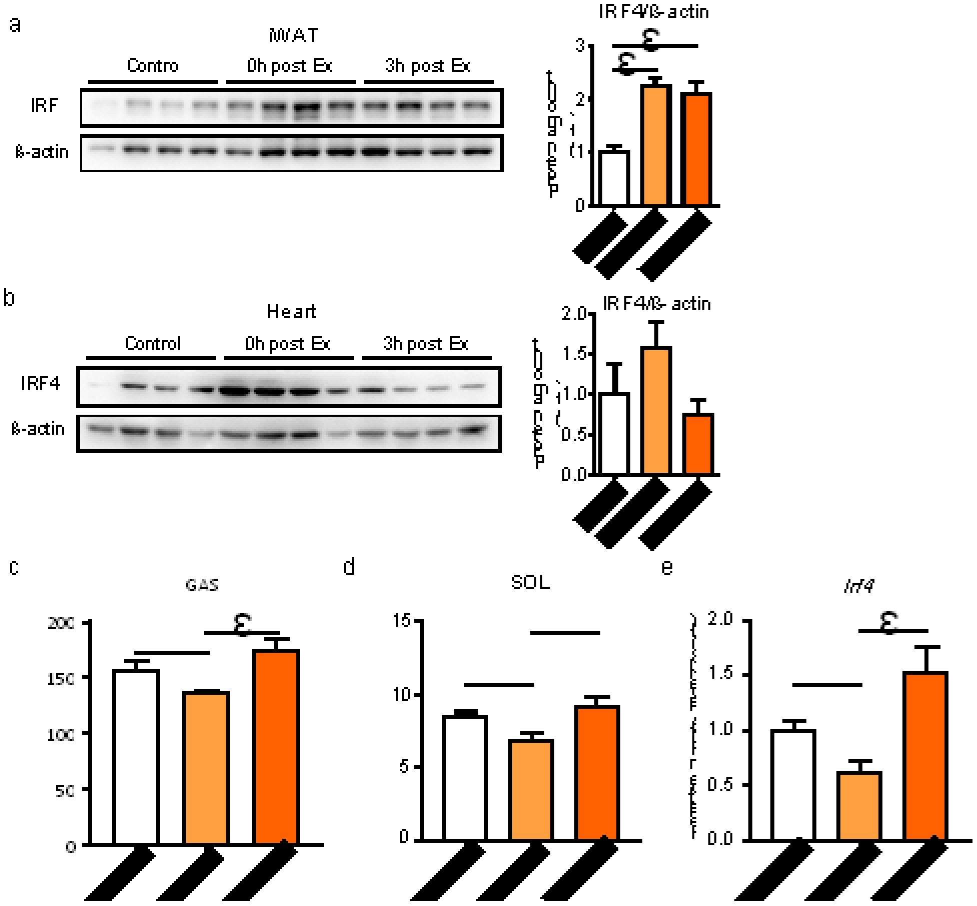

Figure S1. The association of skeletal muscle IRF4 with exercise in mice with hindlimb suspension. a,b Western blot analysis of the expression of IRF4 in inguinal white adipose tissue (iWAT) and heart of WT mice in sedentary, 0h after exercise, and 3h after exercise group. Protein amount was quantified using Image J (n=4, ***p*<0.01, ****p*<0.001). **c,d** The weight of GAS and SOL of WT mice in control, unload, and unload with reload groups (n=4, **p*<0.05, ***p*<0.01). **e** qPCR analysis of Irf4 expression in skeletal muscle of WT mice in control, unload, and reload groups (n=4, **p*<0.05, ***p*<0.01). All results are expressed as means ± SEM.

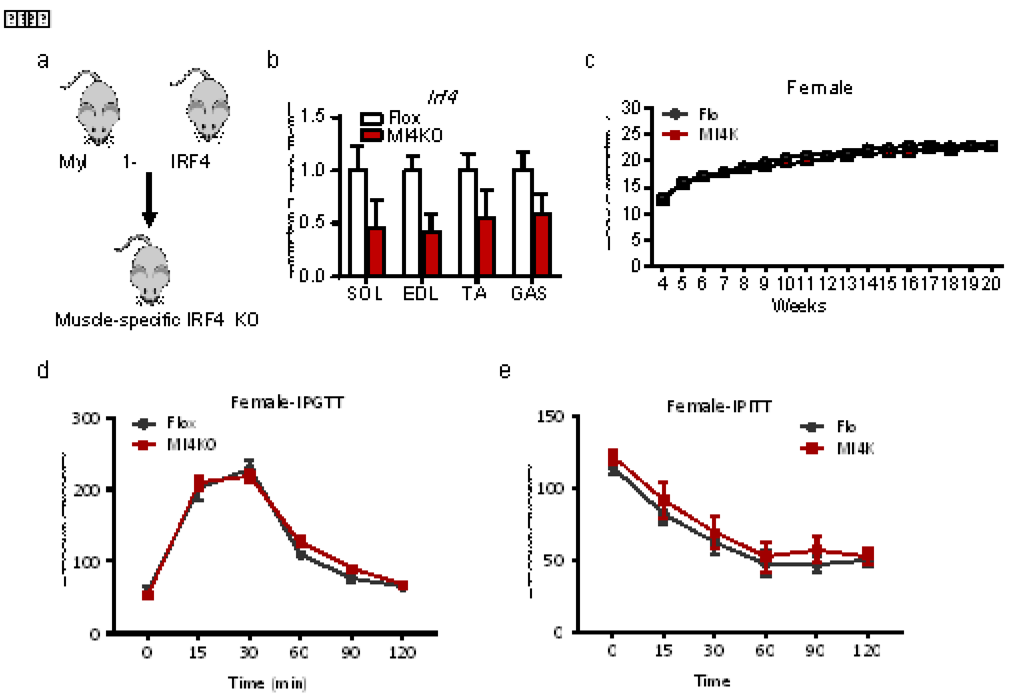

Figure S2. Loss of IRF4 in skeletal muscle shows normal systematic metabolism in female mice. a The muscle-specific IRF4 knockout (MI4KO) mice were generated by crossing Myl1-Cre mice with IRF4 Flox mice. **b** qPCR analysis of Irf4 expression in skeletal muscle of MI4KO and Flox mice (n=6, **p*<0.05). **c** The body weight of female MI4KO and Flox mice on chow diet (n=8-10). **d,e** The glucose tolerance test and insulin tolerance test in female MI4KO and Flox mice on chow diet at the age of 9 weeks and 10 weeks, respectively (n=8). All results are expressed as means ± SEM.

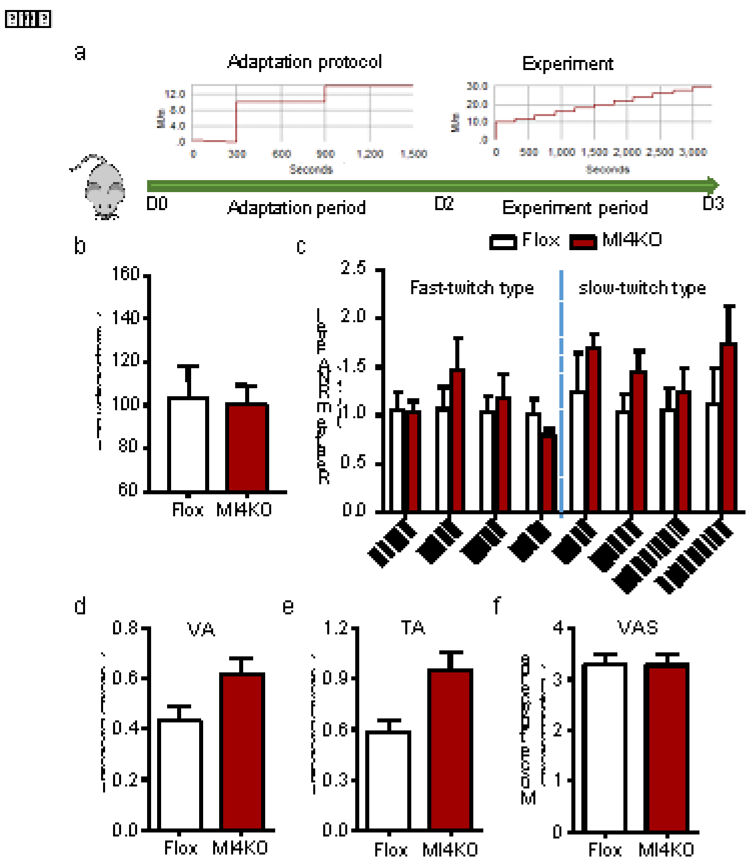

Figure S3. Ablation of IRF4 in skeletal muscle increases the exercise capacity, but not the grip strength. a The protocol of exercise capacity measurement. The mice were adapted to treadmill for 2 days and ran on the third day. **b** The grip strength of male MI4KO and Flox mice on chow diet at the age of 8 weeks (n=10-11). **c** qPCR analysis of the expression of fast-twitch and slow-twitch type genes in skeletal muscle of MI4KO and Flox mice (n=4). **d,e** The glycogen level of VAS and TA in male MI4KO and Flox mice (n = 6, **p*< 0.05). **f** The triglyceride level of VAS in male MI4KO and Flox mice (n=8). All results are expressed as means ± SEM.

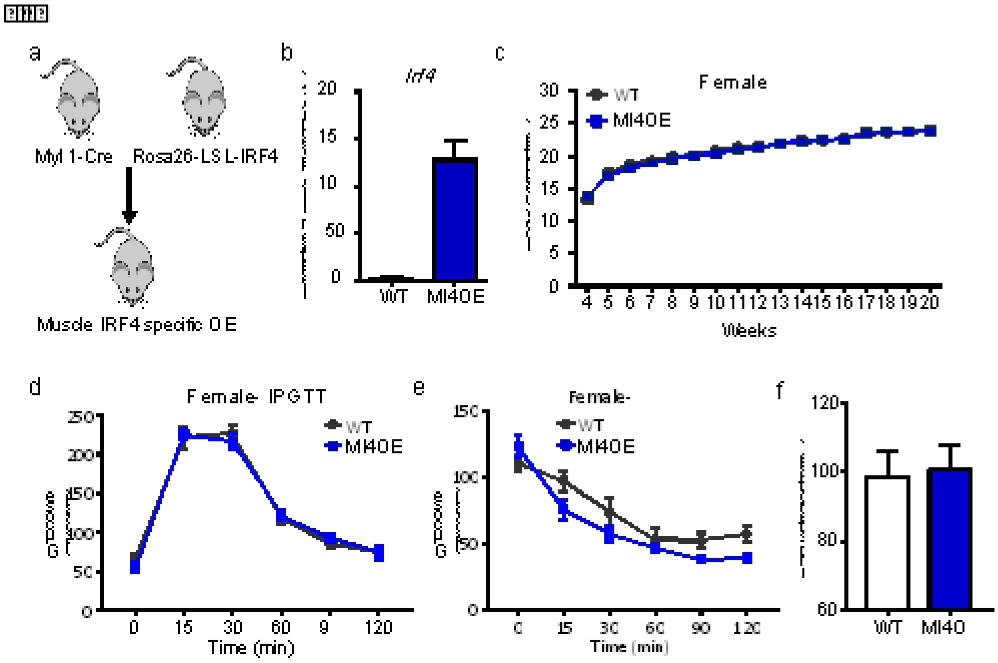

Figure S4. The phenotype of muscle-specific IRF4 overexpression mice. a The generation of muscle-specific IRF4 overexpression (MI4OE) mice. **b** qPCR analysis of IRF4 expression in skeletal muscle of MI4OE and WT mice (n=4, **p*< 0.05). **c** The body weight of female MI4OE and WT mice on chow diet (n=10-11). **d,e** The glucose tolerance test and insulin tolerance test on female MI4OE and WT mice on chow diet at the age of 9 weeks and 10 weeks, respectively (n=8). **f** The grip strength of male MI4OE and WT mice on chow diet at the age of 8 weeks (n=10-11). All results are expressed as means ± SEM.

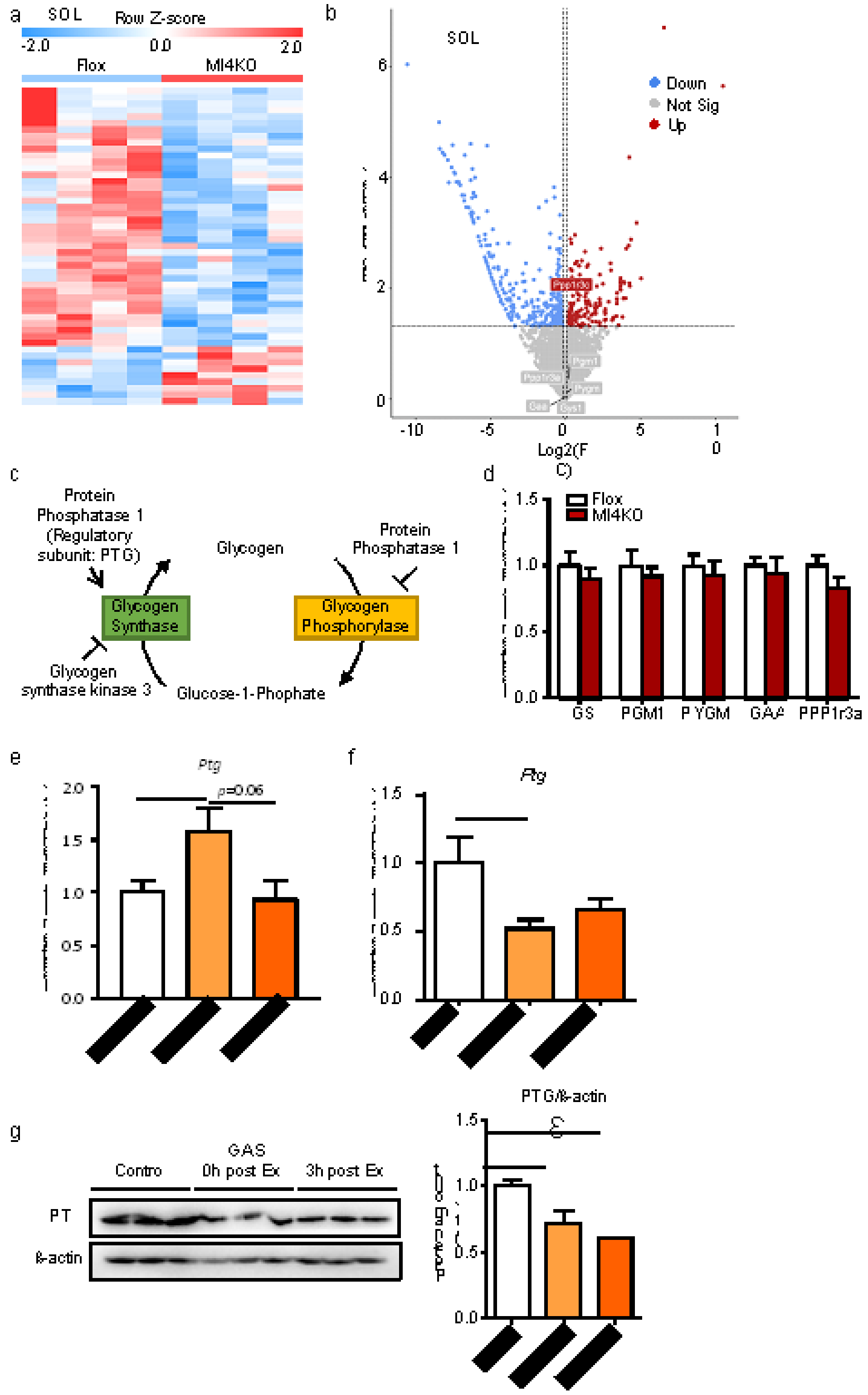
a SOL
Row Z-score
-2.0
0.0
2.0
Flox
MI4KO
b
SOL
Down
Not Sig
Up
Log2(FC)
c
Protein Phosphatase 1 (Regulatory subunit: PTG)
Glycogen
Protein Phosphatase 1
Glycogen Synthase
Glycogen Phosphorylase
Glycogen synthase kinase 3
Glucose-1-Phophate
d
Flox
MI4KO
GS
PGM1
PYGM
GAA
PPP1r3a
e
Ptg
p=0.06
f
Ptg
g
GAS
Contro
0h post Ex
3h post Ex
PT
ß-actin
PTG/ß-actin

Figure S5. RNA-seq of skeletal muscle from MI4KO and Flox mice. a Heat map of differentially expressed genes in SOL of MI4KO and Flox mice (n=4). **b** Volcano plot of differentially expressed genes in SOL of MI4KO and Flox mice (Down: *p* value<0.05 & logFC<-0.58; Up: *p* value<0.05 & logFC>0.58). Genes that relative to glycogen metabolism were labeled. **c** Glycogen metabolism pathway. **d** qPCR analysis of genes relative to glycogen metabolism in skeletal muscle of MI4KO and Flox mice (n=7-8). **e,f** qPCR analysis of PTG expression in skeletal muscle of WT mice in HLS and exercise groups. **g** Western blot analysis of the expression of PTG in skeletal muscle of WT mice in sedentary, 0h after exercise, and 3h after exercise group. Protein amount was quantified using Image J (n = 3, *$p < 0.05$). All results are expressed as means ± SEM.

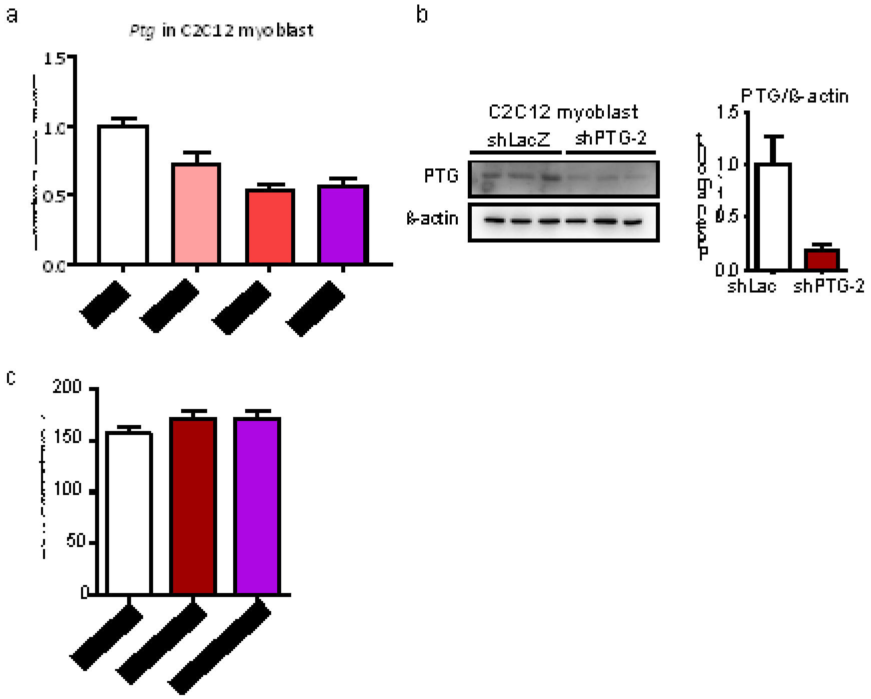

Figure S6. Knockdown of PTG in cells and mice. a qPCR analysis of PTG expression in C2C12 myoblast (n = 3, *$p < 0.05$). **b** Western blot analysis of the expression of PTG in C2C12 myoblasts. Protein amount was quantified using Image J (n = 3, *$p < 0.05$). **c** The weight of GAS

in male Flox and MI4KO mice with or without AAV-shPTG injection (n=7-8). All results are expressed as means ± SEM.

Table S3. Top 31 genes changed in both soleus and gastrocnemius

Number	Gene symbol
1	1700001022Rik
2	Alb
3	Amigo3
4	Apoc1
5	Arhgap26
6	Arrdc2
7	Bcl6b
8	C2cd4c
9	Ccl21a
10	Cdc14a
11	Cish
12	Cyp2e1
13	Fkbp5
14	Fmod
15	Foxo1
16	Gbp10
17	Gdf11
18	Irf4
19	Kif1a
20	Mt1
21	Mt2

22	Nnmt
23	Odf3l2
24	Ppp1r3c
25	Rbp4
26	Retnla
27	Selp
28	Slc39a14
29	Syt12
30	Trf
31	Zfp91Cntf

Table S4. Sequence of primers and shRNA

Gene	Species	Forward primer	Reverse primer
Irf4	Mouse	CAGGACTACAATCGTGAGGAGG	GCACATCGTAATCTTGTCTTCCA
Tbp	Mouse	CCCCTTGTACCCTTCACCAAT	GAAGCTGCGGTACAATTCCAG
Ppp1r3c/PTG	Mouse	TGATCCATGTGCTAGATCCACG	ACTCTGCGATTTGGCTTCCTG
Actn3	Mouse	AACAGCAGCGGAAAACCTTCA	GGCTTTATTGACATTGGCGATTT
Myh1	Mouse	GCGCAACGTGGAAGCTATCAAGGGTCTG	GATCTTCACATTTTGCTCATCTTTTGGTCACT
Myh4	Mouse	CCTGGAACAGACAGAGAGGAGCAGGAGAG	GTGAGTTCCTTCACTCTGCGCTCGTGC
Mstn	Mouse	AGTGGATCTAAATGAGGGCAGT	GTTTCCAGGCGCAGCTTAC
Myh7	Mouse	GCTGGAAGATGAGTGCTCAGAG	TCCAAACCAGCCATCTCCTCTG
Myh7b	Mouse	GGTGTTACACCAACTACGCTGC	TCTGCTGTCCACAAAGGTGTCG
Myoglobin	Mouse	GTGCCTGGAACATCCCTAAT	TGAGAGGCTGTTCTCTCTGC
Troponin1	Mouse	CATGGTTGCACCGTGCTCACAG	GAGCCCATGGCTCAGCCCTG
shPTG-1	Mouse	AAAAGCATTTCTTATCACGCTAATGTT	CACCGCATTTCTTATCACGCTAATGC
		CGCATTAGCGTGATAAGAAATGC	GAACATTAGCGTGATAAGAAATGC
shPTG-2	Mouse	AAAAGCAATTCTAGATCTGTATTGCTT	CACCGCAATTCTAGATCTGTATTGCC

shPTG-3	Mouse	CGGCAATACAGATCTAGAATTGC<br>AAAAGCTATAGAAGCTATAGCTACTTT<br>CGAGTAGCTATAGCTTCTATAGC	GAAGCAATACAGATCTAGAATTGC<br>CACCGCTATAGAAGCTATAGCTACTC<br>GAAAGTAGCTATAGCTTCTATAGC
